# Birth of clones of the world’s first cloned dog

**DOI:** 10.1038/s41598-017-15328-2

**Published:** 2017-11-10

**Authors:** Min Jung Kim, Hyun Ju Oh, Geon A Kim, Erif Maha Nugraha Setyawan, Yoo Bin Choi, Seok Hee Lee, Simon M. Petersen-Jones, CheMyong J. Ko, Byeong Chun Lee

**Affiliations:** 1 Department of Theriogenology and Biotechnology, College of Veterinary Medicine, Seoul National University, 1 Gwanak-ro, Gwanak-gu, Seoul, 08826 Republic of Korea; 2 Department of Small Animal Clinical Sciences, College of Veterinary Medicine, Michigan State University, 736 Wilson Road D-208, East Lansing, MI 48824 USA; 3 Department of Comparative Biosciences, College of Veterinary Medicine, University of Illinois at Urbana-Champaign, 3806 VMBSB, MC-002, 2001 South Lincoln Avenue, Urbana, Illinois 61802 USA

## Abstract

Animal cloning has gained popularity as a method to produce genetically identical animals or superior animals for research or industrial uses. However, the long-standing question of whether a cloned animal undergoes an accelerated aging process is yet to be answered. As a step towards answering this question, we compared longevity and health of Snuppy, the world’s first cloned dog, and its somatic cell donor, Tai, a male Afghan hound. Briefly, both Snuppy and Tai were generally healthy until both developed cancer to which they succumbed at the ages of 10 and 12 years, respectively. The longevity of both the donor and the cloned dog was close to the median lifespan of Afghan hounds which is reported to be 11.9 years. Here, we report creation of 4 clones using adipose-derived mesenchymal stem cells from Snuppy as donor cells. Clinical and molecular follow-up of these reclones over their lives will provide us with a unique opportunity to study the health and longevity of cloned animals compared with their cell donors.

## Introduction

The 10^th^ birthday of the world’s first cloned dog, Snuppy, was celebrated in April 2015, but he died just 13 days later. Snuppy was a symbol of a revolutionary breakthrough in dog cloning achieved using somatic cell nuclear transfer (SCNT). Cloning mammalian species from adult cells was first achieved with the birth of Dolly the sheep in February 1997^1^, which triggered efforts to develop cloning of dogs. The first attempt, the Missyplicity Project was started in August 1997 by a team of Texas A&M University scientists with a private fund of $3.7 million dollars aiming to clone a dog called Missy. However, the project ended without success. The failure of the dog cloning attempt was in sharp contrast with the successful cloning of mice^2^, cattle^3^, pigs^4^, goats^5^, rabbits^6^, and cats^7^. What made dog cloning challenging was certain unique aspects of the reproductive process in canids compared to most other mammals. These included the fact that canids are monoestrus, they ovulate at the metaphase I stage, and there is heterogeneity of oocyte maturation. We overcame these features that had thwarted earlier cloning attempts and in 2005 using *in vivo* matured oocytes produced “Snuppy”, a clone derived from adult cells of a male Afghan hound, Tai^8^. Time magazine named Snuppy as one of the most amazing inventions of the year^9^.

There has been debate from the start of cloning as to whether clones would have normal health, growth, reproduction and longevity. Although birth defects have been reported in some cloned puppies, clones born without defects showed normal growth and reproduced successfully^10^. Growth characteristics including body weight, height and bone length, as well as hematological characteristics including complete blood counts and serum chemistries were all within normal reference ranges. Additionally, reproductive characteristics including hormonal levels, gonad formation, and gamete production were also within normal ranges. Based on these findings, dog cloning has been utilized for a number of purposes including conservation of endangered species, the cloning of companion dogs, the production of clones of service dogs that with outstanding abilites, and the production of transgenic dogs for research purposes^10^. Considering the importance of dog cloning in these area, it is critically important to evaluate the long-term health and life-span of cloned dogs. However, there remains a paucity of scientific reports comparing the health and life-span of clones and their cell donors. Here we compare the long-term health and life-span of Snuppy with his cell donor and report on the recloning of Snuppy, providing a unique opportunity to further investigate the health and longevity of clones by monitoring these reclones.

## Results and Discussion

The median life-span of domestic dogs ranges between 7 and 15 years, depending on the breeds, with that of the Afghan hound being 11.9 years^11^. Tai, the cell donor for Snuppy, was raised as a companion animal at a home and was euthanized at the owner’s request following the diagnosis of hemangiosarcoma at 12 years of age. On the other hand, Snuppy was raised as a laboratory animal in an animal facility at Seoul National University and died while undergoing anticancer treatment at 10 years of age. Despite the different housing environment, Snuppy lived a life that is similar to its cell donor Tai and did not exhibit any health problems until being diagnosed with T-cell lymphoma at 9 years of age. Cancer is common in aged dogs of all breeds, accounting for 27% of all deaths in purebred dogs^11^ and 45% of deaths in dogs over 10 years of age^12^. Although cancer is a multifactorial disease, there is a breed predisposition for certain types of cancers suggesting a genetic component^13^. For example, Irish water spaniels and flat-coated retrievers are reported to have the highest cancer-driven mortality with more than 50% of each breed dying from cancer^11^. The Afghan hound is reported to have a mortality rate from cancer of 30.8%, with osteosarcoma being overrepresented^11^. For now, it is not known whether a potential genetic predisposition that might have led to hemangiosarcoma in Tai was involved in the development of T-cell lymphoma in Snuppy; any predisposition for cancer in Tai’s family cannot be determined because all the littermates of Tai died due to accidents before they were 8 years of age, at ages before most cancers commonly develops.

The suggestion that cloned animals might have a reduced lifespan was made when Dolly died at 6 years of age, and the early death of cloned mice was reported^14^. However, successful serial recloning in mice over multiple generations^15^ as well as a normal healthy age span of recloned sheep derived from Dolly^16^ were subsequently reported. Up to now, except for Dolly and Cumulina (the first cloned mouse), the lifespan of the world’s first cloned animals of other species has not been reported and there are a lack of reports comparing the lifespan of clones with their cell donors. Longevity studies using farm animals is suggested to be somewhat problematical due to the occurrence of accidents, accident-associated infections, inappropriate management, diarrhea, pneumonia, etc.^17^. In fact, Dolly died due to one of those problems, and thus, longevity of the world’s first cloned sheep could not be compared to her cell donor. This makes comparing Snuppy with the cell donor and investigation of the longevity of the recloned dogs especially valuable.

**Figure 1:**
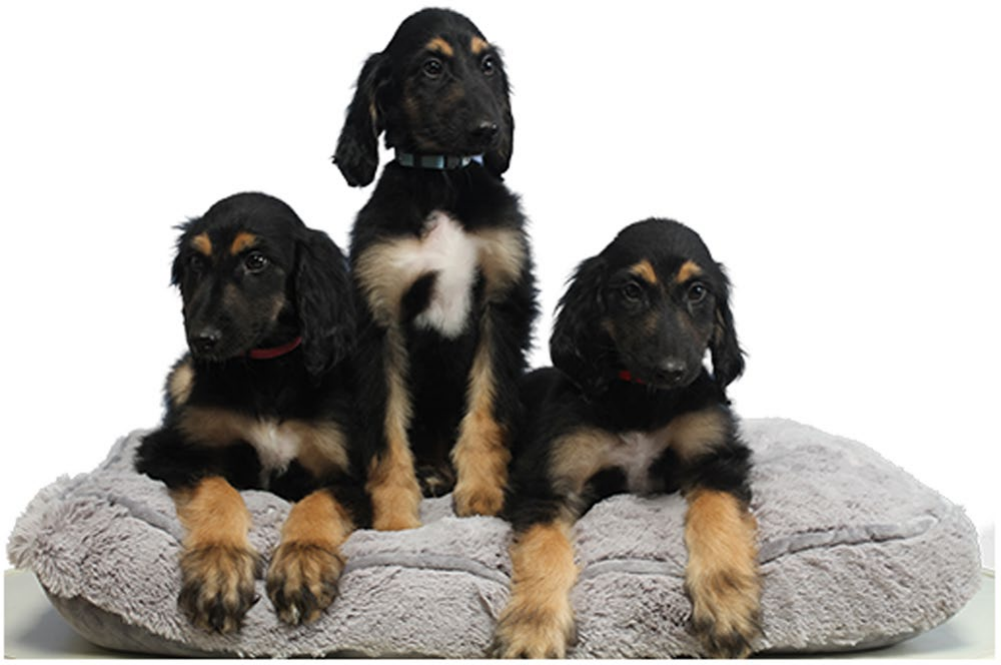
The three surviving reclones at 2 month of age. They were dervived by SCNT of adipose-derived mesenchymal stem cells (ASCs) taken from Snuppy at five years of age.

To immortalize the milestone achievement of cloning a dog and to provide the genetic resources for further research, we recloned Snuppy. Adipose-derived mesenchymal stem cells (ASCs) taken from Snuppy at five years of age were used for the recloning. A total of 120 oocytes matured *in vivo* were recovered from female donors by flushing their oviducts, and the ASCs developed from Snuppy were injected into the perivitelline space of 112 enucleated oocytes, of which 97 couplets were fused and activated. In keeping with our previous report that dog ASCs cultured with Dulbecco’s Modified Eagle Medium (DMEM) increased the fusion rate of dog to cow interspecies SCNT^18^, ASCs cultured with DMEM produced an improved mean fusion rate of 86.6% (range 75.0% –100.0%) from our previously achieved mean rate of 67.2% (range 46.7–85.0%, unpublished data) in dog to dog SCNT. A total 94 reconstructed SCNT embryos were transferred to the oviducts of seven naturally synchronized recipient dogs. Interestingly, all three recipients, that received 13, 13, and 14 cloned embryos on the same day, were confirmed to be pregnant by ultrasonography after 26 days, and four clones were born by Caesarian section 59 days later. Pregnancy and delivery rates for the reclones were 42.9% (3 dogs from 7 recipients) and 4.3% (4 clones from 94 embryos), respectively, which compared favorably with the results when Snuppy was cloned of 2.4% and 0.2%, respectively. All the reclones were healthy when delivered and had normal morphology. The birth weights were 410, 480, 490, and 500 g, which is within the normal range for this breed. Unfortunately, one reclone died 4 days after birth due to severe diarrhea for which the etiology was not identified. Perinatal mortality in dogs is relatively common and has been reported to occur in between 13.3% and 24.6% of litters^19,20^. So early neonatal death of one puppy from our litter of reclones is similar to what would commonly occur in a regular litter of puppies. At the time of writing this report, the other three reclones are 9 months of age, of similar weights and remain healthy (Fig. 1). Microsatellite analysis confirmed that they were reclones of Snuppy (see Supplementary Table 1).

## Conclusion

The world’s first cloned dog, Snuppy had a life-span that was very similar to that of his somatic cell donor. He did not exhibit any notable health problems until the development of cancer which was also diagnosed in his cell donor at a similar age. Three healthy reclones of Snuppy are alive, and as with Snuppy we do not anticipate that the reclones will go through an accelerated rate of aging or will be more prone to develop diseases than naturally bred animals. With the data from Tai and Snuppy in hand, we are excited to follow the long-term health and aging processes of these second generation of clones and work with them to contribute to a new era of studying longevity of cloned canines and given the history of both Tai and Snuppy they may also provide potential insights into the development of cancer.

## Methods

### Ethics statement

All experiments involving animals, methods and protocols were approved by the Committee for Accreditation of Laboratory Animal Care and the Guideline for the Care and Use of Laboratory Animals of Seoul National University (SNU-160602-6). All methods and protocols were carried out in accordance with the relavant guidelines and regulations. All chemicals were purchased from Sigma Chemical Company (St. Louis, MO, USA) unless otherwise specified.

### Donor cell preparation

Adipose tissues were collected from inguinal region of Snuppy when it was five years of age, and transferred to the laboratory as eptically. From these tissues, mesenchymal stem cells were isolated as previously described^21^. Briefly, the adipose tissues were washed with phosphate buffered saline (PBS), minced with scissors in the dish, and digested with 1 mg/ml collagenase I (Gibco, Carlsbad, CA, USA) under gentle agitation for 60 min at 37 °C. After filtering through a 100 μm cell strainer and centrifugation, the cell pellet was resuspended in 5% fetal bovine serum-containing RKCM (Rmedia-stemcell, Seoul, Korea). After centrifugation, the supernant was removed and the cell fraction cultured overnight at 37 °C, 5% CO_2_, in RKCM medium. The cell cultures were maintained in the same media over four to five days until confluence, and then were stored at passage 0 in liquid nitrogen until later characterization or donor cell preparation. *In vitro* differentiation of canine ASCs  were confirmed at passage 2. The cryopreserved cells were thawed, cultured prior to SCNT and then retrieved from the monolayer by trypsinization.

### Oocyte collection and somatic cell nuclear transfer (SCNT)

Collection of *in vivo* matured oocytes and SCNT were performed based on the initial dog cloning study^8^ with small modifications. Ovulation of a female dog was determined by serum progesterone concentration, and a midline laparotomy was performed under general anesthesia approximately 72 hours after the predicted time of ovulation. After exteriorizing an ovary, a flushing needle was inserted into the opening of the infundibulum and then tied in place using 2–0 nylon (Blue Nylon; Ailee, Busan, Korea). An intravenous catheter was inserted into the caudal portion of the oviduct, and ovulated oocytes were collected by flushing the oviduct with tissue culture medium-199 (TCM-199; Invitrogen, Carlsbad, CA, USA) supplemented with 10 mM HEPES, 2 mM NaHCO_3_, 5 mg/mL of BSA (Invitrogen), and 1% (v/v) penicillin-streptomycin. After removal of cumulus cells by repeated pipetting, mature oocytes were selected and used for SCNT. The denuded oocytes were enucleated using micromanipulators (Nikon Narishige, Tokyo, Japan) under an inverted microscope equipped with epifluorescence. An ASC with homogenous cytoplasm was selected and injected into the perivitelline space of an enucleated oocyte. The oocyte-cell couplets were fused with two pulses of direct current of 72 V for 15 s each using an Electro-Cell Fusion apparatus (NEPA GENE, Chiba, Japan), and fused couplets were activated by calcium ionophore and 6-demethylaminopurine.

### Embryo transfer and parturition

Right after activation of the reconstructed embryos, they were surgically transferred into the oviduct of a recipient dog as described earlier^8^. After exteriorizing the left oviduct of a recipient by laparotomy, cloned embryos in HEPES-buffered TCM-199 were place in the ampullary portion of the oviduct using a 3.5 Fr Tom Cat Catheter (Sherwood, St Louis, MO) introduced through the opening of the infundibulum of the oviduct. Only naturally 1-day-advanced asynchronous or synchronous recipients^22^ were used in this study. Pregnancy diagnosis was performed 26 days after embryo transfer by ultrasound using a SONOACE 9900 (Medison, Seoul, Korea) ultrasound scanner with 7.0 MHz linear-array probe. The number of fetuses was confirmed 45 days after the embryo transfer, and pregnancy was monitored as previously described until parturition^23^.

### Parental analysis for genotyping

Genomic DNA was extracted from nuclear donor fibroblasts of the clone, four recloned dogs, oocyte donors and surrogate recipients to confirm genetic identity. The following seven markers which have been proven for use in cloned dog parentage analysis^24^ were selected: PEZ1, PEZ3, PEZ6, PEZ8, FH2010, FH2054 and FH2079. The isolated genomic DNA samples were dissolved in 50 μl lx TE buffer and used for investigation of the seven microsatellites. The microsatellites were amplified by polymerase chain reaction with fluorescently labeled (FAM, HEX, and NED) locus-specific primers and amplicon length analysis performed using an automated DNA sequencer (ABI 373; Applied Biosystems, Foster City, CA) with proprietary software (GeneScan and Genotyper; Applied Biosystems).

## Electronic supplementary material

Dataset 1
