# Supplementary material for: Birth of clones of the world’s first cloned dog
Source: Sci Rep. 2017 Nov 10;7:15235. doi: 10.1038/s41598-017-15328-2 (PMC5681657; doi:10.1038/s41598-017-15328-2)
Supplement: Supplementary file 1 — Dataset 1 [file 41598_2017_15328_MOESM1_ESM.doc]

**Birth of clones of the world’s first cloned dog**

Min Jung Kim^1^, Hyun Ju Oh^1^, Geon A Kim^1^, Erif Maha Nugraha Setyawan^1^, Yoo Bin Choi^1^, Seok Hee Lee^1^, Simon M Petersen-Jones^2^, CheMyong Jay Ko^3^, Byeong Chun Lee^1, *^

^1^ Department of Theriogenology and Biotechnology, College of Veterinary Medicine, Seoul National University, 1 Gwanak-ro, Gwanak-gu, Seoul 08826, Republic of Korea

^2^ Department of Small Animal Clinical Sciences, College of Veterinary Medicine, Michigan State University, 736 Wilson Road D-208, East Lansing, MI, 48824, USA

^3^ Department of Comparative Biosciences, College of Veterinary Medicine, University of Illinois at Urbana-Champaign, 3806 VMBSB, MC-002, 2001 South Lincoln Avenue, Urbana, Illinois 61802, USA

^*^ Correspondence to: Byeong Chun Lee. Department of Theriogenology and Biotechnology, College of Veterinary Medicine, Seoul National University, 1 Gwanak-ro, Gwanak-gu, Seoul 08826, Republic of Korea.

E-mail: [bclee@snu.ac.kr](mailto:bclee@snu.ac.kr).

**Supplementary table 1.** Microsatellite genotyping of Snuppy, Re-Snuppies, oocyte donors and recipients.

| **Marker** | **Clone** | **Reclone** | | | | **Oocyte donors** | | | | | **Recipient** | | |
| --- | --- | --- | --- | --- | --- | --- | --- | --- | --- | --- | --- | --- | --- |
|  | **Snuppy** | **1** | **2** | **3** | **4** | | **A** | **B** | **C** | **D** | **E** | **F** | **G** |
| PEZ1 | 135/135 | 135/135 | 135/135 | 135/135 | 135/135 | | 135/139 | 135/135 | 135/139 | 135/139 | 135/141 | 135/135 | 135/139 |
| PEZ3 | 128/131 | 128/131 | 128/131 | 128/131 | 128/131 | | 116/134 | 125/131 | 128/134 | 125/128 | 125/125 | 143/143 | 128/137 |
| PEZ6 | 180/180 | 180/180 | 180/180 | 180/180 | 180/180 | | 172/176 | 188/188 | 188/188 | 184/192 | 184/184 | 184/184 | 184/184 |
| PEZ8 | 236/236 | 236/236 | 236/236 | 236/236 | 236/236 | | 224/224 | 224/240 | 228/228 | 228/240 | 224/228 | 224/228 | 228/240 |
| FH2010 | 232/232 | 232/232 | 232/232 | 232/232 | 232/232 | | 232/236 | 228/240 | 236/240 | 236/240 | 232/236 | 240/240 | 228/240 |
| FH2054 | 168/176 | 168/176 | 168/176 | 168/176 | 168/176 | | 156/156 | 152/152 | 152/156 | 156/172 | 152/152 | 152/156 | 152/172 |
| FH2079 | 272/272 | 272/272 | 272/272 | 272/272 | 272/272 | | 276/280 | 272/276 | 272/276 | 272/276 | 272/276 | 276/276 | 272/276 |
